# From the Editor’s desk

**DOI:** 10.4103/0974-1208.69330

**Published:** 2010

**Authors:** Kamini A Rao

**Affiliations:** Editor-in-Chief, Journal of Human Reproductive Sciences E-mail: drkaminirao@gmail.com

The current issue of the journal has two review articles. The first one is by Pandey *et al*., from the University of Aberdeen, UK, which concludes that obesity is associated with lower fertilization rates, poor quality embryos and higher miscarriage rates. Weight loss in these women improves their reproductive outcomes but the authors stress on the importance of a gradual and sustained weight loss program as against crash dieting which is almost always detrimental.

The second review article touches on the complex subject of sperm maturation, capacitation, and fertilization. The understanding of this at a molecular level may help us lead to new and better techniques for enhancing fertility, identifying and treating certain forms of male subfertility, and preventing conception. Another study in this issue on the correlation of human sperm centrosomal proteins with fertility demonstrates lower centrosome protein expression in oligoasthenozoospermic males as compared to normozoospermic men which may be a possible cause of their reduced fertility status.

In assisted reproduction, selection of the right embryos for transfer remains the key factor to success, more so in frozen embryo transfer cycles. This issue has an article which shows that transfer of embryos cleaved during overnight culture after thawing led to significantly higher pregnancy rates than transfer of those without any cleavage. So far, more importance has been given to the selection of the embryos prior to cryopreservation; however, studies such as this show that post-thaw selection of embryos also plays an equally important role.

Ovarian Hyper Stimulation Syndrome (OHSS) is the bane of any COH program. Many strategies have been used to prevent OHSS such as cancelling the cycle, coasting, modifying the method of ovulation triggering, administration of albumin or plasma expanders, etc. Till date, there is no fool proof method that exists to avoid OHSS. Today, a few papers have been published on the use of bromocryptine in reducing the severity of OHSS. Vinita Sherwal in her study has shown the usefulness of dopamine agonist, bromocriptine, in reducing the incidence of severity of OHSS in high-risk patients without affecting the pregnancy rates. However, the optimum dose of bromocriptine needs to be worked out through larger Randomized Controlled Trials (RCT).

Four interesting case reports have been published in this issue. One case report is of a woman with removal of endometrial osseous metaplasia, resulting in spontaneous conception. The others are of septate uterus with hypoplastic left adnexa with cervical duplication and longitudinal vaginal septum, successful expectant management of a tubal heterotopic pregnancy, and a case of the clinical significance of hyperprolactinemia with normal serum prolactin levels.

Now for some news unrelated to the journal. I am really proud to announce that the Karnataka chapter of ISAR will soon come into force to facilitate all members to actively participate in state-level professional activities, organize conferences and generally promote the cause of infertility in Karnataka. Orissa, Maharashtra, Uttar Pradesh, Madhya Pradesh, Chhatisgarh and few more other states have already floated local chapters. It is really good to know that ISAR is spreading its tentacles and will be reaching out the smaller towns and cities of India.

